# Cost-effectiveness analysis for clinicians

**DOI:** 10.1186/1741-7015-10-10

**Published:** 2012-02-01

**Authors:** Suzanne R Hill

**Affiliations:** 1 World Health Organization, 20 Ave Appia, Geneva 27, Switzerland

## Abstract

In a climate of economic uncertainty, cost effectiveness analysis is a potentially important tool for making choices about health care interventions. Methods for such analyses are well established, but the results need to be interpreted carefully and are subject to bias. Making decisions based on results of cost-effectiveness analyses can involve setting thresholds, but for individual patients, there needs to be disaggregation of benefits and harms included in a quality adjusted life year to ensure appropriate consideration of benefits and harms as well as personal preferences and circumstances.

## Background

The current economic news is bad. Expenditure on health care is under scrutiny in every country and with predictions of another economic crash comes pressure on the funding of health care systems - systems that, according to the World Health Organization http://www.who.int/topics/health_systems/en/ are meant to deliver quality service to all people, when and where they need them.

At the same time, new products - pharmaceuticals, diagnostic tests, new technologies - continue to be developed and launched. Health care systems are under pressure to pay for all of the potential hope that these provide, sometimes with good reason, sometimes without. This is where cost-effectiveness analysis comes in.

## Discussion

In 1990, Detsky and Naglie [1] published one of the first guides to cost-effectiveness analysis for clinicians. As they described it, cost-effectiveness analyses compare the costs and outcomes for a new intervention with an existing alternative treatment, strategy or intervention. The two questions such analyses aim to answer are: how much does the new intervention cost compared with current practice and is it more effective, and if so, how much more? The results of these analyses are presented as additional cost per additional benefit, that is, additional dollars per unit benefit gained. This is the incremental cost effectiveness ratio (ICER). Benefits can be expressed as different outcomes: as change in a physiological or biological unit of measurement (for example, change in glycated hemoglobin (HbA1c)), difference in lives gained (Life Year Gained, LYG), or differences in quality adjusted life years (QALY). The choice of the outcome depends on what data exist that can be used in the evaluation and whether a mathematical model can be developed that simulates the course of the disease or condition and its effect on the quality of life. Inputs into these models are usually derived from multiple sources, including clinical trials, observational studies and routine databases, to name just a few. The degree of uncertainty associated with the primary source of information will contribute to the uncertainty about the results from the model.

The results of cost-effectiveness analyses should provide the same information as that used for making decisions about any purchasing choice in every day life. If a new strategy or potential purchase is more effective and costs less than the currently available option, it is almost certainly worth doing. Likewise, if the new strategy is less effective and more expensive, no one is likely to buy it. However, the more usual outcome of a cost effectiveness analysis of a health technology is that the new technology may be more effective, but costs more. The judgment that then needs to be made is whether the benefit obtained is worth the cost and how certain we can be about that assessment.

A quick literature search suggests that if the number of publications is an indicator of public interest, then answering these questions is a key concern of health care policy makers. A random selection of topics for such analyses range from the evaluation of different strategies for lipid lowering [2] to the use (or not) of radiological evaluation of bronchiolitis in children [3]; from the use of surgical options in the treatment of chronic back problems [4] to the use of pharmacogenetic tests to manage anticoagulation [5]. Health technology assessment authorities, organizations making coverage decisions, and clinical guideline producers are the 'consumers' of these publications. There has been considerable debate about the reliability of the versions published in journals. Udvarhelyi *et al. *[6] published one of the first critiques of published cost-effectiveness analyses in 1992, pointing out the problems with published analyses compared to optimal methods and since then many authors have identified problems with bias in evaluations [7-10]. As the analyses are often conducted by commercial entities, the results may favor the product owned by the sponsor [11-15] but there are also more simple problems regarding the calculations. Translating a change in blood pressure into survival gains and then quality adjusting them requires translation, extrapolation and mathematical modeling and can go wrong anywhere in the equations. In the end, the estimate of the size of the effect of the intervention, however expressed, is one of the main determinants of the outcome of the model, so it needs to be examined closely.

To ensure robust analyses and reliable results, those interested in conducting them have defined methods and checklists that specify the gold standard for methods for cost-effectiveness evaluations [16-18]. However, there are still challenges in applying the results of an analysis to decisions. For decisions that apply to a public health care sector, 'thresholds' for 'acceptable' cost-effectiveness estimates, that is, a specified value for the ICER, have been suggested by the World Health Organization (WHO) and used by organizations such as the National Institute for Health and Clinical Excellence (NICE) in the UK, and have also been hotly debated [19-22]. One crucial problem with thresholds is how to set them and how to apply them. Values and preferences of communities are much more difficult to determine or quantify, but may have greater weight in choices about health care, particularly in tax-payer-funded systems.

One challenge in interpreting and applying the results from cost-effectiveness analyses is the difference between the value of an intervention for the population and the value for an individual patient. For a clinician, the response and cost and benefit from a particular intervention for an individual patient may not be well represented by a cost-effectiveness ratio from a population. Ioannidis and Garber [23] point out that cost-effectiveness estimates, particularly those that express the results as incremental cost per QALY, combine several different outcomes in the 'QALY', including estimates of benefits and harms that may mean quite different things for different individuals. Differences in background health, a person's aversion to risk, or their personal circumstances are likely to have a significant effect on how an individual interprets the tradeoff between benefits, harms and costs, and thus chooses between treatment strategies. The solution suggested by Ioannidis and Garber is to 'individualize' the ICER by presenting a per person benefit in QALYs expressed as days, a 'per person cost' as well as evaluating subgroup responses within populations to allow estimates of variance within the 'average' ICER, to match an individual to a population that may be more or less likely to benefit. It would be interesting to test this approach in real life - personalized medicine based on personalized health economics. A new discipline to develop, to satisfy the needs of individual patients and clinicians, as well as payers and politicians simultaneously!

## Conclusion

Methods for cost effectiveness analysis are well established, but published analyses need to be interpreted carefully. For clinicians, disaggregating the benefits and harms incorporated into estimates of QALY may inform judgments about use of health interventions for individual patients.

## Abbreviations

HbA1C: glycated hemoglobin; ICER: incremental cost effectiveness ratio; LYG: life year gained; NICE: National Institute for Health and Clinical Excellence; QALY: quality adjusted life year; WHO: World Health Organization.

## Competing interests

The authors declare that they have no competing interests.

## Authors' contributions

The author is the sole author of this manuscript, and prepared and approved the final version.

## Author information

The author was a staff member of the World Health Organization and is now Chair of the Australian Pharmaceutical Benefits Advisory Committee. She alone is responsible for the views expressed in this publication and they do not necessarily represent the decisions, policy or views of the World Health Organization or the Department of Health and Aging, Australia.

## Pre-publication history

The pre-publication history for this paper can be accessed here:

http://www.biomedcentral.com/1741-7015/10/10/prepub
